# Retroperitoneal cecal volvulus: a complication of a rare internal hernia – a case report

**DOI:** 10.1097/MS9.0000000000001540

**Published:** 2024-01-04

**Authors:** Imad Kamaleddine, Magdalena Popova, Theresa Angles, Manuela Neese, Beate Brinkmann, Erik Volmer, Marc-André Weber, Georg Lamprecht, Clemens Schafmayer, Ahmed Alwali

**Affiliations:** a Department of General, Visceral, Vascular, Thoracic, and Transplantation Surgery; b Department of Medicine II, Division of Gastroenterology and Endocrinology; c Institute of Diagnostic and Interventional Radiology, Pediatric Radiology and Neuroradiology, Rostock University Medical Center, Rostock, Germany

**Keywords:** case report, cecal volvulus, foramen of Winslow hernia, internal hernia, intestinal obstruction

## Abstract

**Introduction and importance:**

The foramen of Winslow hernia (FWH) is a rare type of internal hernia. In one-third of cases, the cecum was found in the lesser sac. More rarely, the herniated cecum might be volvulated, which represents 1–1.5% of the causes of intestinal obstruction. Once diagnosed, surgical reduction and/or resection of the nonviable herniated bowel is crucial for a positive outcome.

**Case presentation:**

The authors report a case of retroperitoneal cecal volvulus that complicated FWH in a patient with a history of laparoscopic cholecystectomy.

**Clinical discussion:**

A delay in the diagnosis is associated with high morbidity and even higher mortality. Because of lacking a consensus, the treatment of FWH depends on the team’s surgical experience.

**Conclusion:**

Reporting this case will help us to keep in mind this differential diagnosis while treating patients in our daily practice.

## Case description

Highlights

- The Foramen of Winslow hernia represents a rare type of internal hernias.
- A cecal volvulus is a rare case of intestinal obstruction representing 1–1.5% of all cases.
- A radiological exam, usually a computed tomography scan, should be performed.
- A high clinical suspicion should be raised once a part of the bowel is found in the lesser sac.
- Surgery is the treatment of choice with nowadays no consensus or guideline.
- Most of the published cases are case reports.

**Figure 1:**
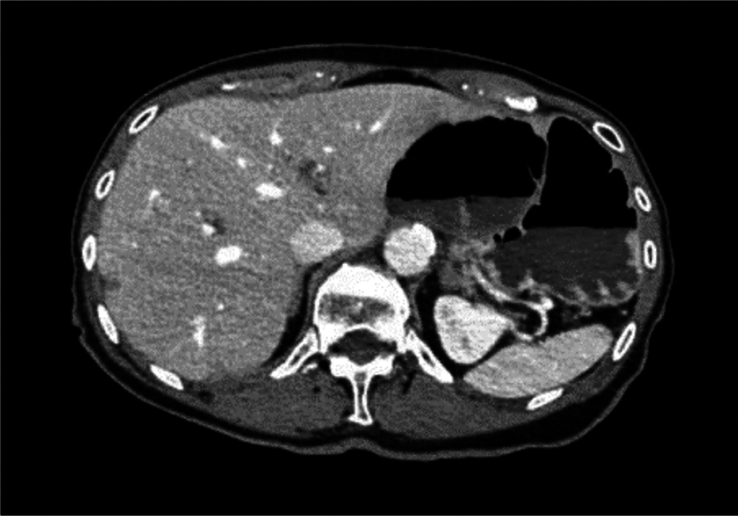
Contrast-enhanced computed tomography scan in the portal-venous phase of the upper abdomen showing the dilated colon next to the stomach.

**Figure 2:**
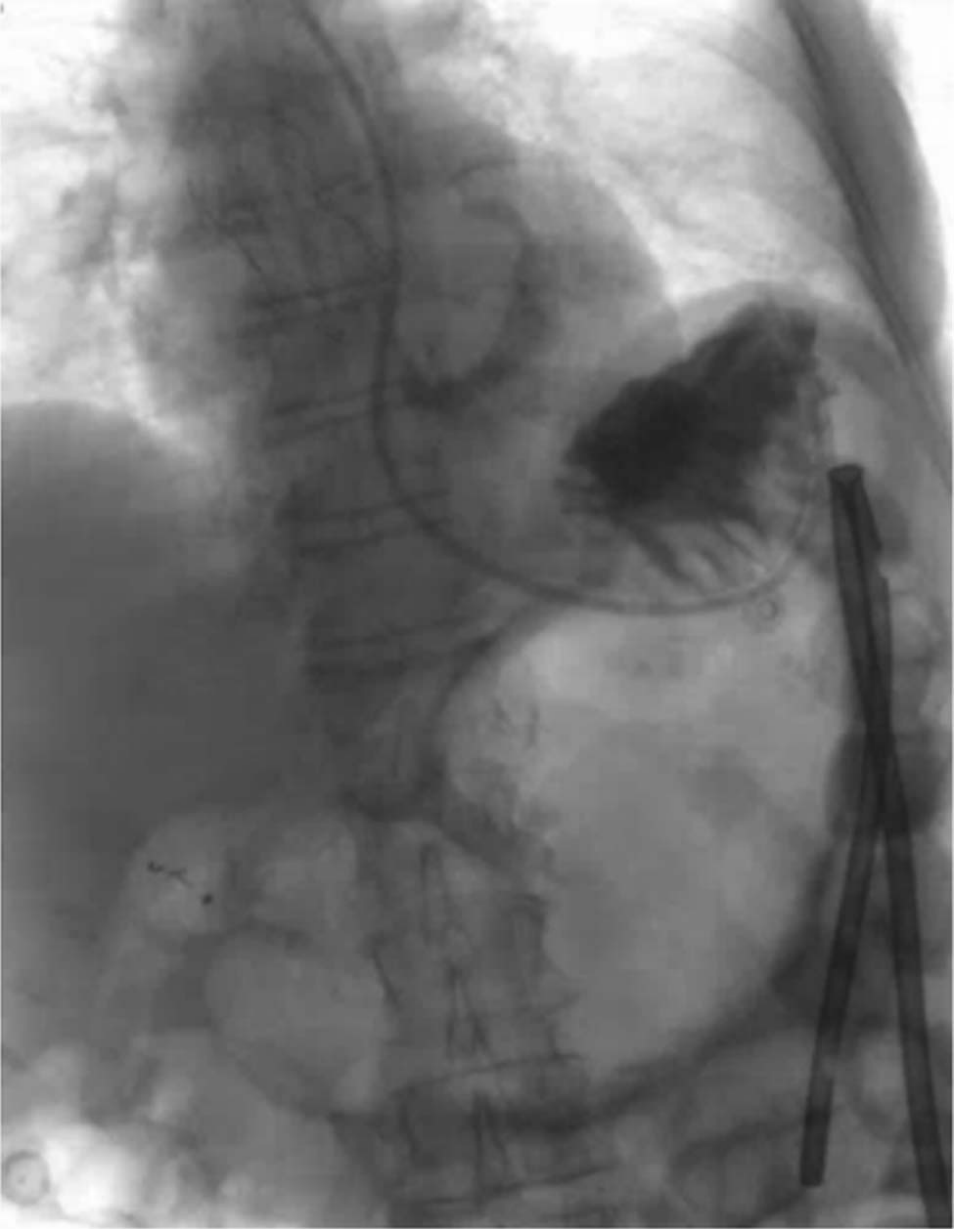
Fluoroscopy after colposcopy and contrast administration through the NGT: neither the NG-tube nor the colonic decompression tube stain or reach the large gas filled lumen arguing against it being the transverse colon or the stomach.

**Figure 3:**
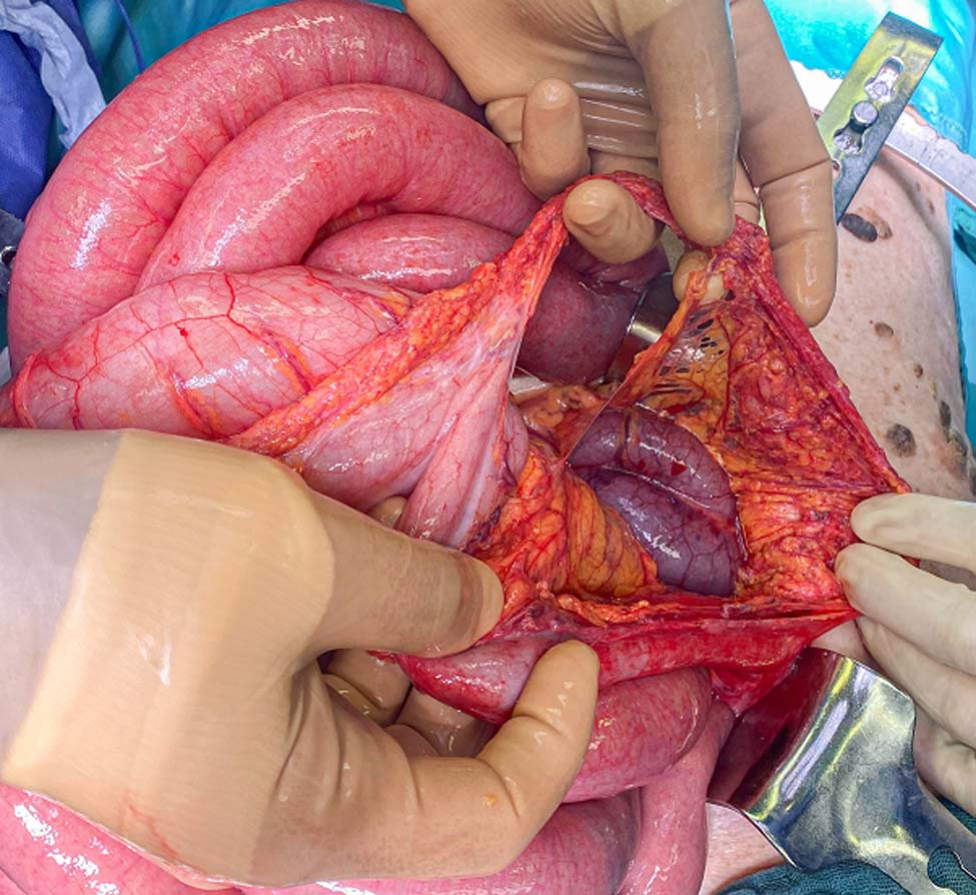
Herniated cecum in the lesser sac.

**Figure 4:**
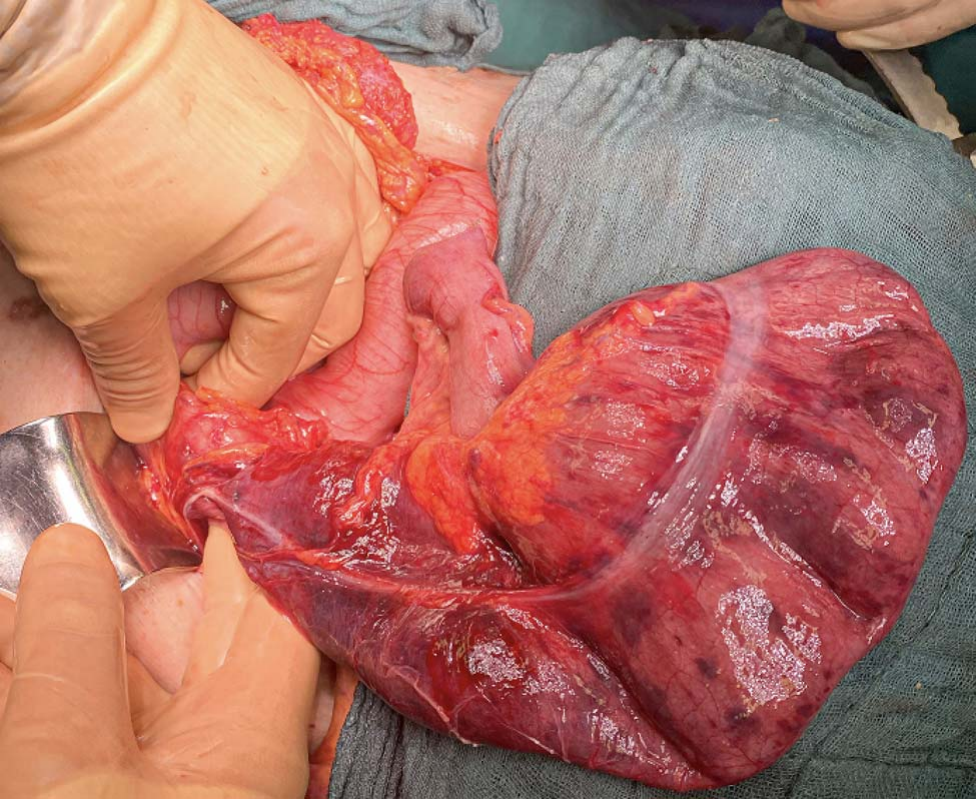
Ischemic cecum after decompression.

The SCARE criteria were followed in reporting this case report^1^. We present the case of an 83-year-old woman, who was admitted through the emergency department few hours after the sudden onset of thoracic and upper abdominal pain associated with vomiting. The pre-existing conditions include: laparoscopic cholecystectomy, history of percutaneous coronary intervention and drug-eluting stent implantation in the right coronary artery, and left anterior descending artery/first diagonal branch (D1), transcatheter aortic valve implantation for high-grade aortic stenosis and mitral/tricuspid valve insufficiency, renal insufficiency (stage G3a), type II diabetes mellitus, arterial hypertension, dyslipoproteinemia, history of basal cell carcinoma, and restless legs syndrome. The physical examination showed a cachectic patient with no jugular vein congestion or peripheral edema on auscultation, a regular heart rhythm with no murmurs, an unremarkable pulmonary examination, and a distended abdomen with no signs of peritonitis. Upon admission, a synthetic opioid, Piritramid 7.5 mg was intravenously (iv) administered. The patient was initially pain-free but experienced a pain recurrence shortly after that. Computed tomography (CT)-scan of both thorax and abdomen with with iv contrast-enhancement excluded a possible aortic dissection and showed signs of intestinal transit disorder in the left upper abdomen and a distended transverse colon without signs of mesenteric ischemia (Fig. 1). Subileus was diagnosed and conservative treatment was initiated, including gastric and rectal tube installation, fluid therapy, correction of hypokalemia, proton pump inhibitor, analgesic, and antibiotic therapy. Under this treatment, the patient showed no remarkable improvement. Colonoscopy and gastroscopy under fluoroscopy were then performed. Due to stool soiling, only the transverse colon could be reached but was not dilated as suggested in the CT. However, a tumoral stenosis was ruled out and an intestinal decompression tube was inserted. The concomitant gastroscopy also showed no signs of malignancy or stenosis. After repositioning the nasogastric (NG) tube, water-soluble contrast medium (Peritrast 400 mg, Köhler Pharma) was administered through both tubes and an abdominal radiography was performed. Unexpectedly, neither the contrast via the NG-tube nor the colonic decompression tube stained or reached the the large, air-filled, coffee bean-shaped structure in the upper left abdomen (Fig. 2). In the following hours, the rectal decompression tube was dislocated, the patient’s abdomen remained distended and painful. Due to these unclear findings a new interdisciplinary discussion took place. The physical examination showed diffused abdominal pain with signs of local peritonitis in the epigastrium. In the context of the upon mentioned findings and the clinical condition of the patient there was a clear indication for exploratory laparotomy. An informed consent was obtained. A median laparotomy was performed. The intraoperative findings showed ascites with omental adhesions (postcholecystectomy), dilated small bowels and a slim colon transverse and left colon. The small intestine was followed from the Treitz band and surprisingly was fixed in the right upper quadrant. The Lesser sac was widely opened and just then the diagnosis was clear: cecal volvulus after herniation through the foramen of Winslow (Fig. 3). The cecum was then decompressed by a purse-string suture and appendectomy. The cecal wall was ischemic (Fig. 4), and as a consequence a right hemicolectomy with side-to-side anastomosis was performed. The postoperative course was uneventful. A regular diet was tolerated and the patient was discharged on the ninth postoperative day in good condition.

## Discussion

The first reported case of the foramen of Winslow hernia (FWH) was published in 1834 through Philippe-Frederic Bladin as an incidental postmortem finding. Since then, ~200 such cases have been reported worldwide (Table 1). FWH accounts for 8% of internal hernias and 0.08% of all hernias^37^. In one-third of the cases, the hernia included the cecum and ascending colon^113^. Among the possible causes of predisposition are: an enlarged foramen of Winslow, an abnormally long small bowel mesentery, persistence of the ascending mesocolon that allows marked mobility of the bowel, and an elongated right hepatic lobe^4,57,114^. On the other hand, cecal volvulus accounts for 1% of large bowel obstruction causes^113^. This manifestation has two forms with the more common one involving an axial twist of the ileum, cecum, and proximal ascending colon around its mesentery. The other form, called cecal bascule, accounts for 10% of the cases and involves the folding of the cecum upward toward the hepatic^115^. Cecal volvulus, once untreated, is usually associated with a high mortality rate that reaches 48%^109^. This happens usually due to vascular involvement and delayed diagnosis^45,114^.

**Table 1:** Summary of all the previous published cases

N	Authors	Type of publication	Year of publication	Number of cases	History of cholecystectomy	Herniated organ
1	JE Engstad^2^	Case report	1919	1	No	Small bowel
2	WG McKenzie and D Wood^3^	Case report	1925	1	No	Small bowel
3	JM Erskine^4^	review	1967	90	No	—
4	JM Erskine^5^	Case report	1967	1	No	Cecum
5	CD Haynes *et al*.^6^	Case report	1968	1	No	—
6	Dainko *et al*.^7^	Case report	1970	1	No	Ileum, right colon
7	W Morioka *et al*.^8^	Case report	1970	1	No	Cecum
8	UD Campbell^9^	Case report	1971	1	No	Cecum
9	IJ Malter *et al*.^10^	Case report	1973	1	No	Cecum
10	RK Harned *et al*.^11^	Case report	1974	1	No	Ileum and Cecum
11	L Riedler *et al*.^12^	Case report	1975	1	No	Ileum, right and transverse colon
12	R Ohkuma *et al*.^13^	Case report	1977	1	No	Small bowel
13	DJ Cohen *et al*.^14^	Case report	1982	1	No	Cecum
14	B Sorin *et al*.^15^	Case report	1982	1	No	Cecum
15	CP Valenziano *et al.* ^16^	Case report	1987	1	Yes	Ascending colon
16	J Delamarre *et al*.^17^	Case report	1988	1	No	Cecum
17	TL Tran *et al*.^18^	Case report	1989	2	No	1-right colon 2-Ileum
18	W Lemish *et al*.^19^	Case report	1989	1	No	Cecum
19	JW Greve *et al*.^20^	Case report	1990	1	No	Ileum and Cecum
20	JJ Tjandra *et al*.^21^	Case report	1991	1	No	Right colon
21	S Beizig *et al*.^22^	Case report	1992	1	No	Ascending colon
22	MR Schuster *et al*.^23^	Case report	1992	1	No	Cecum
23	E Senati *et al*.^24^	Case report	1994	1	No	Right colon
24	V Evrard *et al*.^25^	2 Case reports	1996	2	No	1-right colon 2-Ileum
25	J Nagahori^26^	Case report	1996	1	No	Gallbladder
26	CC Chung *et al*.^27^	Case report	1997	1	No	Small bowel
27	Y Saida *et al*.^28^	Case report	2000	1	No	Small bowel
28	MJ Orseck *et al*.^29^	Case report	2000	1	No	Right flexure
29	TD Samson *et al*.^30^	Case report	2001	1	No	Cecum
30	PB Rich *et al*.^31^	Case report	2002	1	No	Right colon
31	Antao *et al*.^32^	Case report	2005	1	No	Ileum
32	SS Forbes *et al*.^33^	Case report	2006	1	No	Ascending colon
33	LM Pernice *et al*.^34^	Case report	2006	1	No	Transverse colon
34	G Da Costa *et al*.^35^	Case report	2007	1	No	Cecum
35	CE Koh *et al*.^36^	Case report	2007	2	No	1-Gallbladder 2-Cecum
36	AB Osvaldt *et al*.^37^	Case report	2007	1	No	Small bowel
37	D Kanellos *et al*.^38^	Case report	2008	1	No	Ileum, right colon
38	A Mbovo *et al*.^39^	Case report	2008	1	No	Ileum
39	J Izumi *et al*.^40^	Case report	2009	1	No	Gallbladder
40	M. Iribarren Diaz *et al*.^41^	Case report	2009	1	No	Ascending colon
41	Rajeswaran *et al*.^42^	Case report	2009	1	No	Cecum
42	K Ray *et al*.^43^	Case report	2009	1	No	Cecum
43	LH Webb *et al*.^44^	Case report	2009	1	No	Ascending colon
44	AR Azar *et al.* ^45^	Case report	2010	1	No	Ileum and Cecum
45	K MacDonald *et al*.^46^	Case report	2010	1	No	Ileum, right colon
46	AD Clough *et al*.^47^	Case report	2011	1	No	Transverse colon
47	Stahlfeld KR *et al*.^48^	Case report	2011	1	No	Small bowel
48	E Van Dael *et al*.^49^	Case report	2011	1	No	Right colon
49	SF Powell-Brett *et al*.^50^	Case report	2012	1	No	Cecum
50	RL Smith *et al*.^51^	Brief report	2012	1	No	Ileum, right colon
51	CR Gonzalez *et al*.^52^	2 Case reports	2013	2	No	1-Small bowel 2-Cecum, Ileum
52	Lin *et al*.^53^	Case report	2013	1	No	Ileum
53	A May *et al*.^54^	Case report	2013	1	No	Small bowel
54	K Numata *et al*.^55^	Case report	2013	1	No	Gallbladder
55	V Patel *et al*.^56^	Case report	2013	1	No	Cecum
56	CA Puig *et al.* ^57^	Case report	2013	1	No	Right colon
57	T Yamashiro *et al*.^58^	Case report	2013	1	No	Ileum
58	T Dissanayake *et al*.^59^	Case report	2014	1	No	Cecum
59	P Sikiminywa-Kambale *et al.* ^60^	Case report	2014	1	No	Cecum
60	T Makarawo *et al*.^61^	Case report	2014	1	No	Cecum
61	J Ryan *et al*.^62^	Case report	2014	1	No	Right colon
62	CL Tee *et al*.^63^	Case report	2014	1	No	Small bowel
63	S Nazarian *et al*.^64^	Case report	2015	1	No	Small bowel
64	CR Harnsberger *et al.* ^65^	Case report	2015	1	No	Cecum and Ileum
65	M Ozsoy *et al*.^66^	Case report	2015	1	No	Cecum and Ileum
66	PN Brandao *et al*.^67^	Case report	2016	1	No	Transverse colon
67	LE Duinhouwer *et al*.^68^	Case report	2016	1	No	Ascending colon
68	R Daher *et al*.^69^	Case report	2016	1	No	Cecum and Ileum
69	S Garg *et al*.^70^	Case report	2016	1	No	Cecum and Ileum
70	LS Kirigin *et al*.^71^	Case report	2016	1	No	Small bowel
71	V Sobek *et al*.^72^	Case report	2016	1	No	Cecum
72	E Leung *et al*.^73^	Case report	2016	1	No	Small bowel
73	G Tse *et al.* ^74^	Case report	2016	1	No	Cecum
74	HG Cho *et al*.^75^	Case report	2017	1	No	Small bowel
75	Y Ichikawa *et al*.^76^	Case report	2017	1	No	Small bowel
76	J Nguyen *et al*.^77^	Case report	2017	1	No	Cecum
77	J Patel *et al*.^78^	Case report	2017	1	No	Cecum
78	BW Deschner *et al*.^79^	Case report	2018	1	No	Cecum
79	A Haddad *et al*.^80^	Case report	2018	1	No	Small bowel
80	YJL Jansen *et al*.^81^	Case report	2018	1	No	Cecum
81	K Shek *et al*.^82^	Case report	2018	1	No	Cecum
82	P Downs *et al*.^83^	Case report	2018	1	No	Ileum, right and transverse colon
83	S Fujihata *et al*.^84^	Case report	2018	1	No	Small bowel
84	F Ayoob *et al*.^85^	Case report	2019	1	No	Ileum and Cecum
85	P Charters *et al.* ^86^	Case report	2019	1	No	Cecum
86	D Moris *et al*.^87^	Review	2019	15	No	—
87	YA Mahnashi *et al*.^88^	Case report	2019	1	No	Right colon
88	M Azer *et al*.^89^	Case report	2020	1	No	Right colon
89	C Buisset *et al*.^90^	Case report	2020	1	No	Right colon
92	YM Cho *et al*.^91^	Case report	2020	1	No	Cecum
93	E Ristiyanto *et al*.^92^	Case report	2020	1	No	Small bowel
94	M Sammut *et al*.^93^	Case report	2020	1	No	Cecum
95	I Sravya *et al*.^94^	Case report	2020	1	No	Small bowel
96	S Akhtar *et al*.^95^	Case report	2021	1	No	Cecum
97	PKBSC Bandara *et al*.^96^	Case report	2021	1	No	Small bowel
98	EA Karlsen^97^	Case report	2021	1	No	Small bowel
99	AM. Williams *et al*.^98^	Case report	2021	1	No	Cecum
101	N Naqeeb *et al.* ^99^	Case report	2021	1	Yes	Cecum
102	S Honma *et al*.^100^	Case report	2021	1	No	Small bowel
103	D Chandhrasekhar *et al*.^101^	Case report	2022	1	No	Ileum, right colon
104	SL Carpenter *et al*.^102^	Case report	2022	1	No	Cecum
105	AL Titan *et al*.^103^	Case report	2022	1	No	Small bowel
106	A Kharkhash *et al*.^104^	Case report	2022	1	No	Cecum
107	EL Monteiro *et al*.^105^	Case report	2022	1	No	Ileum, right colon
108	H Honda *et al*.^106^	Case report	2022	1	No	Right colon
109	Y Huang *et al*.^107^	Case report	2022	1	No	Small bowel
110	SM Mansoor *et al*.^108^	Case report	2022	1	No	Cecum
111	A Perabo *et al.* ^109^	Case report	2022	1	No	Cecum
112	V Tatagari *et al*.^110^	Case report	2022	1	No	Ascending colon
113	E Mulkey *et al*.^111^	Case report	2022	1	No	Cecum
114	HWL de beaufort *et al*.^112^	Case report	2023	1	No	Small bowel

The initial symptoms of FWH are usually nonspecific and can include both upper abdominal and chest pain^86^. The diagnosis should be expected once on radiological examinations an air-filled structure is seen in the left upper quadrant. However, this is achieved preoperatively in only 10% of cases^116^. An urgent surgical management involves reduction of the herniated bowel and might be followed by cecopexy and/or closure of the foramen of Winslow. Once bowel ischemia is present, resection should be performed^57,60^. Both open and minimal invasive surgeries have been reported^65,74^.

In our case, after ruling out cardiorespiratory distress, the patient was treated conservatively for subileus. Failure of this therapy and the persisting presence of the air-filled coffee bean structure between both stomach and colon on the fluoroscopy with the developing signs of local peritonitis led to the surgical intervention. The initial clinical examination showed no signs of peritonitis and the CT-scan interpreted the dilated colon in the left upper quadrant as transverse colon and excluded mesenteric ischemia. We began a conservative therapy and the surgical treatment was delayed for 48 h. Ferguson *et al*. in 2008 reviewed the literature for cases of intestinal volvulus as a possible postcholecystectomy complication. Among the 12 published cases, three involved an intraperitoneal cecal volvulus^117^. Interestingly, retroperitoneal cecal volvulus (in the lesser sac through FWH) was also reported twice as a possible complication of laparoscopic cholecystectomy^16,99^. Although it is just speculation, to our knowledge, this case is the third reported case of the rare cecal volvulus after herniation through the foramen of Winslow and a history of cholecystectomy.

## Conclusion

This case demonstrates that even with suitable diagnostic imaging, the initial diagnosis of FWH is not always easy. It also proves the significance of maintaining a strong level of clinical suspicion, especially for the rarest cases. A consensus on the management of this rare clinical manifestation is lacking. However, good clinical suspicion and prompt surgical management are essential for a positive outcome.

## Ethical approval

An ethical approval was not required. A written informed consent was obtained from the patient for publication of this case report with any accompanying images.

## Consent

The authors testify the patient privacy maintenance. Written informed consent was obtained from the patient for publication of this case report and accompanying images. The authors ensure that all the figures/photos are suitably anonymised with no patient information or means of identifying the patient.

## Sources of funding

No funding was provided for this case.

## Author contribution

I.K.: operated on the patient and proposed the writing of the manuscript; C.S.: took the role of supervision; I.K., M.P., and A.A.: wrote the preliminary version of the manuscript, prepared the images, and regulated the necessary files; T.A. and M.N.: assisted at the operation; G.L. and B.B.: performed the endoscopic exams; E.V. and M.A.W.: performed the radiological exams. All authors have read and approved the manuscript.

## Conflicts of interest disclosure

The authors have no conflicts of interest to declare.

## Research registration unique identifying number (UIN)

As a case report, no registration of research is needed.

## Guarantor

Imad Kamaleddine and Clemens Schafmayer.

## Data availability statement

All needed data are available upon reasonable request.

## Provenance and peer review

The paper was not invited.
